# Biocompatible Materials in Otorhinolaryngology and Their Antibacterial Properties

**DOI:** 10.3390/ijms23052575

**Published:** 2022-02-25

**Authors:** Jakub Spałek, Przemysław Ociepa, Piotr Deptuła, Ewelina Piktel, Tamara Daniluk, Grzegorz Król, Stanisław Góźdź, Robert Bucki, Sławomir Okła

**Affiliations:** 1 Institute of Medical Science, Collegium Medicum, Jan Kochanowski University of Kielce, IX Wieków Kielc 19A, 25-317 Kielce, Poland; g.krol@op.pl (G.K.); stanislaw.gozdz@onkol.kielce.pl (S.G.); buckirobert@gmail.com (R.B.); 2 Department of Otolaryngology, Head and Neck Surgery, Holy-Cross Cancer Center, Artwińskiego 3, 25-734 Kielce, Poland; pr.ociepa@gmail.com; 3 Department of Medical Microbiology and Nanobiomedical Engineering, Medical University of Bialystok, Mickiewicza 2C, 15-222 Bialystok, Poland; piotr.deptula@umb.edu.pl (P.D.); tamara.daniluk@umb.edu.pl (T.D.); 4 Independent Laboratory of Nanomedicine, Medical University of Bialystok, Mickiewicza 2B, 15-222 Bialystok, Poland; ewelina.piktel@wp.pl

**Keywords:** biomaterials, nanomaterials, antimicrobial action, osteosynthesis, tissue engineering, voice prosthesis

## Abstract

For decades, biomaterials have been commonly used in medicine for the replacement of human body tissue, precise drug-delivery systems, or as parts of medical devices that are essential for some treatment methods. Due to rapid progress in the field of new materials, updates on the state of knowledge about biomaterials are frequently needed. This article describes the clinical application of different types of biomaterials in the field of otorhinolaryngology, i.e., head and neck surgery, focusing on their antimicrobial properties. The variety of their applications includes cochlear implants, middle ear prostheses, voice prostheses, materials for osteosynthesis, and nasal packing after nasal/paranasal sinuses surgery. Ceramics, such as as hydroxyapatite, zirconia, or metals and metal alloys, still have applications in the head and neck region. Tissue engineering scaffolds and drug-eluting materials, such as polymers and polymer-based composites, are becoming more common. The restoration of life tissue and the ability to prevent microbial colonization should be taken into consideration when designing the materials to be used for implant production. The authors of this paper have reviewed publications available in PubMed from the last five years about the recent progress in this topic but also establish the state of knowledge of the most common application of biomaterials over the last few decades.

## 1. Introduction

Biomaterial is any substance (other than a drug) or combination of substances, natural or synthetic, that can be used for a period of time, independently or as part of a system which treats, augments, or replaces any tissue, organ, or function of the body [1]. The first application of biomaterial in history is most likely a case that was reported a few centuries after the Common Era of ancient medicine for wound closure. Romans have described urologic catheters, and Aztecs used gold dental fillings [2]. Nowadays, technological progress allows the development of implants that are based on innovative biomaterials. We can classify biomaterials by their applications, material physicochemical properties, or their interactions with the patient’s tissue. The application of biomaterials in modern medicine is very wide, for example, artificial joints, bone grafts, dental implants, cardiovascular stents, artificial lenses, plastic surgery implants, trauma and reconstructive surgery materials, and surgical tools. Due to the variety of biomaterials’ functions, their mechanical properties varied from very hard and stiff to very soft and flexible. According to the biomaterials’ nature, we distinguish the builds of polymers, metals, composites, and ceramics materials. We can also classify biomaterials from their level of interaction with the host tissue as bioinert, bioactive, and bioresorbable [3,4,5]. One of the greatest risks associated with placing an implant within the living tissue of a patient is related to the colonization of the material by opportunistic/pathogenic microorganisms and the formation of a bacterial/fungal or mixed biofilm on an implant’s surface. There are some methods to prevent this process, which are described in Section 4.

### 1.1. Polymers

**Figure 1:**
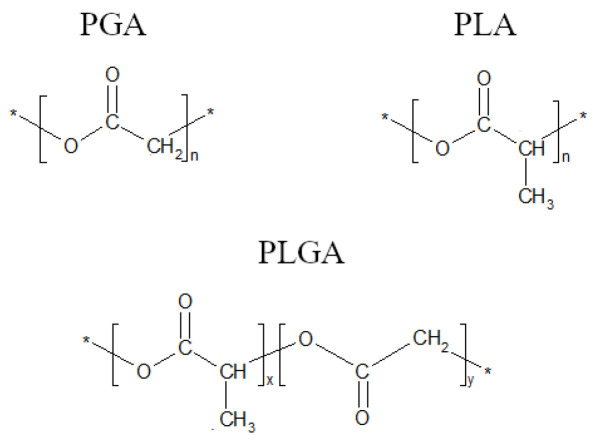
Chemical structures of poly(glycolic acid) (PGA), poly(lactic acid) (PLA), and poly(lactide-co-glycolide) (PLGA). (n: number of repeat units in PLA and PGA; x and y: number of lactic and glycolic units in PLGA, respectively). * another unit of PGA/PLA.

Most common polymers used for the design and fabrication of biomaterials include natural polymers, such as collagen, alginate, or chitosan, and synthetic ones, such as polyethylene, polyethylene terephthalate, and polytetrafluorethylene. Polymers are classified by their permanent (biostable) or temporary (biodegradable) applications. Biostable polymers are used for long-term exploitation. When working with biostable polymers, the main challenge is to prevent the material degradation of the polymer by physiological tissue processes as oxidation of polyether segments in polyurethane at the α-position to the ether-oxygen [6], or the long-term hydrolysis of polyamides [7] or polyethylene terephthalate [7,8]. In most situations, biofilm growth is also a destructive factor for the polymers [9,10]. Biodegradable polymers are used as a base for local drug delivery or as a temporary support for tissue regeneration. These polymers are degraded non-enzymatically by hydrolysis or by specific enzymes [11]. The good biocompatibility makes them a good material for many medical applications [12,13,14,15]. Among the few polymers approved by the FDA, there are poly(glycolic acid) or poly(glycolide) (PGA), poly(lactic acid) or poly(lactide) (PLA), as well as poly(lactic-co-glycolic acid) or poly(lactide-co-glycolide) (PLGA) (Figure 1). 

**Figure 2:**
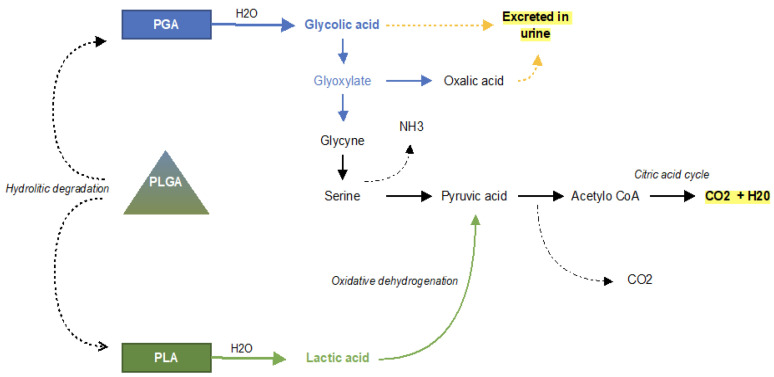
Schematic illustration showing the degradation of PLGA co-polymer and the PGA and PLA monomers. As a result, carbon dioxide and water are finally produced.

In 1970, the US Food and Drug Administration approved these materials for bioresorbable surgical sutures, then, in 1986, the first bioresorbable drug delivery PLGA microspheres were approved [16]. PLGA co-polymers undergo degradation in the way of hydrolysis. The ester bonds are cleaved by the hydrolytic degradation that occurs throughout the whole PLGA microparticle matrix. PLGA degradation into monomers can be divided into three phases. In this process of random chain scission, the polymer divides into the oligomers and finally into soluble monomers. In the first phase, the weight loss and soluble monomer formed are not appreciable, and, in the second phase, there is rapid loss of mass. Once the monomers are formed, they are eliminated by physiological pathways. Lactic acid enters the tricarboxylic acid cycle and is metabolized and eliminated in carbon dioxide and water, while glycolic acid is excreted unchanged by the kidneys or metabolized by the tricarboxylic acid cycle [16,17,18] (Figure 2). So far, 15 products based on PLA/PLGA microparticles have been approved and marketed, and more than 35 products have been successfully developed for medical use [19].

### 1.2. Metals and Metals Alloys

Titanium and its alloys, as well as iron-based alloys and cobalt-based alloys, are commonly used in medicine as materials for all kinds of implants and biomedical constructions, such as orthopedic and bone fracture surgical treatment or as a scaffold of cardiologic self-expanded stents [20,21]. These materials are the most popular metallic biomaterials applied in the broad field of medicine. Stainless steel was applied in the 1920s as a biomaterial, and the first cobalt-based alloy was introduced into dental practice in 1907 [22]. Titanium alloys since the eighties have been model metallic materials used in various types of biomedical constructions [20], unfortunately this material has significant flaws. For many years, toxic alloying agents in titanium alloys, such as aluminum and vanadium, have raised doubts. Studies prove the toxicity of these elements and induction of many diseases after long-term periods of use [23,24]. The main disadvantage of some of these materials is also a very common local inflammatory host reaction or toxicity, which can be decreased by coating them by other biocompatible materials as for example polymers. However, sometimes the local tissue reaction to the material is beneficial, such as stimulating new bones through the use of magnesium alloys in the healing of bone fractures [25]. In recent years, new titanium-based materials with non-toxic additives, such as molybdenum and niobium, which are austenitic steels without the addition of toxic nickel, have been investigated [26,27,28].

### 1.3. Ceramics

Ceramics are a group of biomaterials with very good biocompatibility features. This group generally does not cause an allergic reaction and a cytotoxic effect [29]. But on the other hand these materials are brittle and low impact resistance [30]. Ceramics biomaterials are commonly use in bone replacement, dental and maxillo-facial reconstructions. Aluminum trioxide is still used for dental implants but zirconia, which was introduced as a dental implants material in 1970s is nowadays commonly used because of its similar color to human teeth [31,32].

### 1.4. Composites

Composite materials are made of at least two constituents to decrease their disadvantages. Composite materials are used for bulk soft tissues replacements, space fillers, catheters, ureter prostheses, tendons and ligaments, and vascular grafts. Fiber-reinforced polymer composites are the most commonly used composites in orthopedics [33].

## 2. Methods

Innovative biomaterials for otolaryngology have already been developed. This article aims to review recent publications in this area. Available bibliographies in PubMed were searched, and the latest or the most significant papers, in the authors’ opinions, were quoted. Publications from the last five years (2015–2020) were reviewed in PubMed. Some publications included in this review were published more than five years ago or not found by the queries mentioned below but, in the authors’ opinions, these publications were significant and important. There were 163 articles quoted in this review, following the authors’ research. The following amounts of articles (January 2022) were found after typing some keywords in PubMed: polymers + otolaryngology (828), polymers + head and neck cancers (476), polymers + sinus surgery (263), polymers + head and neck tissue engineering (149), polymers + head and neck implants (125), polymers + cochlear implants (38), and polymers + nasal packing (37).

## 3. Biomaterials Used in Otorhinolaryngology

### 3.1. Cochlear Implants

**Figure 3:**
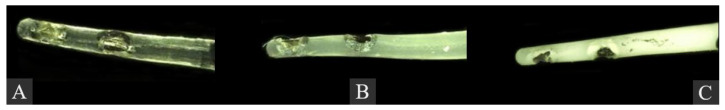
(**A**): pure silicone electrode array without DEX (0%); (**B**): electrode array containing 1% DEX (16 ng/day delivery rate); (**C**): electrode array containing 10% DEX (49 ng/day delivery rate). Adapted from an open-access source: [36].

Cochlear implants are commonly used for the successful treatment of deaf-born children and deafened adults. The idea of this technically advanced method is to implant a cochlear implant’s electrode into the cochlea to allow the electrical stimulation of the auditory nerve [34]. The interaction between the electrode and the auditory neurons is essential for long and effective treatment. The cochlear implant–electrode array consists of platinum–iridium and silicone. Despite its materials’ good biocompatibility, cochlear implants are recognized as foreign bodies. The efficiency of cochlear implants is affected by postoperative connective tissue growth around the electrode array. This results in tissue fibrosis around the electrode, which happens due to fibrocyte migration after the electrode implantation. This process is undesirable because of the increase in impedance that it results in. Glucocorticoids—mainly dexamethasone—are locally applied to decrease fibrosis. One of the methods of its application is drug depot accommodation in the silicon carrier of the electrode [35] (Figure 3). This approach has a more continuous and longer profile of drug release, which is more effective than other methods of dexamethasone application [2,36]. It is desirable to minimize the space between electrodes and auditory neuronal dendrites. For this reason, neurotrophic factors, such as the glial cell-derived neurotrophic factor (GDNF) and the brain-derived neurotrophic factor (BDNF), are also used. Local application of these factors to the cochlear implant electrodes is one of the methods that have a proven positive effect on the anti-fibrosis process, the regeneration of auditory neuron dendrites, and the in vivo preservation of neurons in animals [36,37,38,39]. Lehner et al. have tested a new type of Poly-(D,L-lactic-co-glycolic acid) (PLGA)-based biodegradable implant for intracochlear delivery of drugs on animals to find the appropriate size and mechanical properties, as well as to prove the general feasibility of its administration. They found that the use of Polyethylene glycol (PEG) as an additional excipient was beneficial in two aspects. PEG softens the extrudates and prevents cracking during bending, and PEG accelerates the initial drug release rate so that it matches the desired profile [37]. The release of dexamethasone from the PLGA without PEG was measured at 6.5% within the first week, this then accelerated to reach almost 50% after two weeks and 80% after three weeks. It is connected to initial slow water penetration and the autocatalytic degradation of the polymer [38].

The developed intracochlear drug-loaded implant can be administrated with or without a neuroprosthetic cochlear implant. Some experimental studies also showed the positive application of biodegradable drug delivery systems as a salvage therapy for idiopathic sudden sensorial hearing loss (ISSHL) [37,39]. A carrier of dexamethasone was used along with a PLGA polymer matrix containing a mixture of polymer chains with free and esterified carboxylic end groups without a preservative. The PLGA matrix slowly degrades to lactic acid and glycolic acid. 

### 3.2. Tympanostomy Tube

The implantation of a tympanostomy tube in the eardrum allows the drainage of fluid from the middle ear. This procedure is usually performed in patients with otitis media. Biofilm formation on this device is the main factor of post-tympanostomy complications, such as otorrhea, tube occlusion, and discomfort. There are some studies where authors have faced this problem. In some in vitro studies, the use of a vancomycin coating or a piperacillin-tazobactam coating of the tympanostomy tube were tested with positive promising results [40,41].

Another approach is to change the tube-surface properties of resisting bacterial colonization and biofilm formation. The model implemented by Saidi et. al suggests that the adherence properties of the tube may be more important than antibacterial coatings in terms of the prevention of persistent otorrhea [42]. Jang et al. suggest that the surface modification by an ion bombardment is not enough on its own to resist ciprofloxacin-resistant *Pseudomonas aeruginosa* biofilm formation [43]. Joe et. al designed a novel tympanostomy tube type, i.e., a tympanostomy stent (TS), which had a smooth and minimized surface area to prevent the adherence of biofilm by preserving its own function of drainage. Furthermore, it was coated with TiO_2_. The authors reported the promising outcomes of their study [44]. 

### 3.3. Middle-Ear Prosthesis

The ossicular chain reconstruction of the middle ear may be carried out with either a partial ossicular replacement prosthesis or a total ossicular replacement prosthesis. Implantable middle-ear hearing aids are used in the treatment of mild–moderate, mixed or conductive hearing loss and, in some cases, to treat sensorineural hearing loss [45]. There is a variety of materials used by surgeons for ossicular reconstruction. Some studies indicate that titanium ossicular prostheses are the most popular among surgeons, because of their efficiency in sound transmission and the fact that they are delicate and easy to handle. Titanium is the most lightweight and biocompatible material among all allogenic materials used for ossicular reconstruction [46,47]. Moreover, titanium clip prostheses have proven long-term results in ossiculoplasty [48]. A ceramic (hydroxyapatite) prosthesis commonly used for ossicular reconstruction also exists; however, it has high incidence of extrusion when it is placed in contact with the tympanic membrane [49,50]. A retrospective study comparing hearing and anatomical outcomes after ossicular chain reconstruction with titanium or hydroxyapatite prostheses concluded that both types of prosthesis had satisfactory functional and anatomical results, and no preponderance could be stated, except for the hearing results of partial titanium prostheses [51]. One of the materials used for middle ear surgery is a composite HAPEX (Smith and Nephew). It is composed of 40% synthetic hydroxyapatite (HAp) and 60% high-density polyethylene (HDPE). In a clinical study, HAPEX has proven to be a stable implant/bone bonding material. It was observed that middle-ear prostheses became overgrown by fibrous tissue inside a thin epithelial layer [52]. Problems with extrusion, migration, and reactivity occur with some alloplastic materials, such as Polyethylene, high-density polyethylene sponge (HDPS), polytetrafluoroethylene (PTFE), and Proplast (PTFE–carbon composite), which are also regarded as ossicular prostheses [53]. There are many reports of different artificial materials used in ossicular surgery, but the histocompatibility and long-term outcomes still remain uncertain. Moreover, none of the materials mentioned above possess bactericidal properties [54]. Such activity is looked for as it indicates the same information as the detection of biocompatibility or physical properties. There are some studies about new antimicrobial system, which are fully described in Section 4.

### 3.4. Nasal Packing Materials

**Figure 4:**
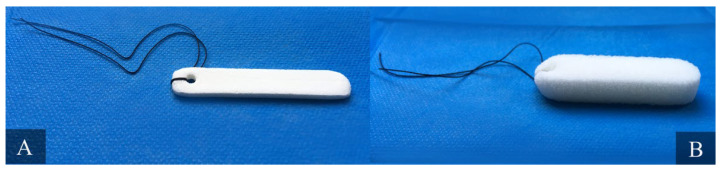
Nonabsorbable nasal packing Merocel (Medtronic Inc., Minneapolis, MN, USA). Panel (**A**) presents compressed, dehydrated sponge. Panel (**B**) shows the sponge decompressed, 30 s after hydration with saline. Material from the authors’ collection.

Endoscopic sinus surgery (ESS), conchotomy, or septoplasty procedures are currently very common surgical treatments. They are often associated with such postoperative complications as nasal bleeding, adhesions, and stenosis, which could be prevented by nasal packing [55]. The efficiency of and the patient’s tolerance for nasal packing products vary [56]. The currently used packing materials can be classified into nonbiodegradable (e.g., vaselinized gauze, Telfapads, cotton-stuffed latex finger cots, Silastic sheeting, Merocel sponges) and biodegradable types (e.g., gel film, MeroGel, hyaluronic acid gels, FloSeal, cellulose gels, Nasopore, NASASTENT) (Figure 4). Nasopore is a fully synthetic biodegradable, fragmenting foam that absorbs water while supporting the surrounding tissue. This process provides local hemostasis by compressing bleeding vessels in the nasal cavity. After several days, it dissolves and can be suctioned from the nasal cavity. Research indicates that biodegradable packing is more comfortable because it results in less pain, bleeding, nasal blockage, and facial edema in the early postoperative period. However, there is no significant difference in the long-term post-operative outcomes of ESS [57]. Wang et al. performed the meta-analysis of 459 articles and concluded that Nasopore (absorbable) is superior to Merocel (non-absorbable), with regard to pain upon removal, bleeding, in situ pain, pressure, and general satisfaction, and equal to Merocel, with regard to nasal obstruction, tissue adhesion, and mucosal healing [58].

### 3.5. Corticosteroid-Eluting Sinus Stents

**Figure 5:**
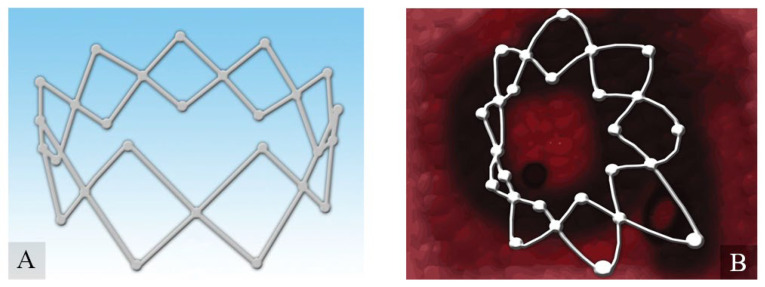
Mometasone-loaded (**A**) spring-like Propel™ sinus implant expands when placed into the sinus mucosa (**B**), thus keeping the middle meatus open and, hence, promoting mucous drainage and wound healing. Adapted from an open-access source: [15].

As nasal packing materials have a positive impact on short-term postoperative outcomes, corticosteroid-eluting sinus stents could increase long-term outcomes. The European Position Paper on Rhinosinusitis and Nasal Polyps (EPOS 2020) guidelines recommend Corticoid-Eluting Sinus Stents as a therapeutic option in patients who have undergone surgical treatment of chronic rhinosinusitis (CRS) to decrease the percentage of re-operations in the future [59]. Recent advancements in bioabsorbable and drug-eluting stents provide an option for improving the long-term outcomes of postoperative endoscopic sinus surgery (ESS). Some patients had sinus neo-ostium stenosis or synechiae formation, middle turbinate (MT) lateralization, after surgery. To prevent this situation and to improve longer-term outcomes, surgeons have used nonabsorbable frontal stents that were typically placed immediately after surgery and removed in the clinic between four and six weeks later because of significant crusting and/or symptomatic pressure. For these reasons, sinus stents have been used sparingly [60]. In 2011, the first corticosteroid-eluting sinus stent (Inter- sect ENT) was approved by the FDA for patients after ethmoid sinus surgery. Then, in 2016 and 2017, the FDA approved similar devices for frontal and maxillary sinus surgeries. [61] Nowadays, there are plenty of bioresorbable stents releasing mainly mometasone for patients with CRS after ESS. There are two models of steroid-eluting sinus implants that have been U.S. Food and Drug Administration (FDA)-approved for use in CRS patients: short-duration Propel family devices (Propel, Propel Mini, Propel Contour; Intersect ENT, Menlo Park, CA, USA) and long-duration Sinuva devices (Intersect ENT, Menlo Park, CA, USA). (Figure 5). 

**Figure 6:**
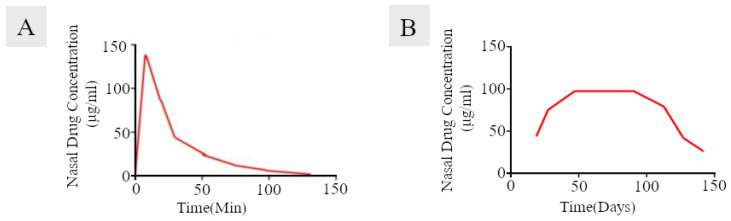
Nasal drug concentration versus time, obtained after administration of nasal sprays and drug-eluting implants. Nasal sprays show rapid clearance of the drug from the nasal mucosa (panel (**A**)) as compared to locally acting implants (panel (**B**)). Adapted from an open-access source: [15].

These novel devices have had promising outcomes in some clinical trials [62,63,64,65]. Drug-eluting nasal implants ensure continuous drug release over longer periods of time to the nasal mucosa, in contrast to the nasal sprays (Figure 6). The corticosteroid was encapsulated in a biodegradable polymer matrix in the form of micro- and nano-particles, and then attached to the biodegradable scaffolds of implants to achieve longer periods of drug release. The most commonly used materials for these implants are biodegradable polymers such as polylactic acid (PLA) and polylactic-co-glycolic acid (PLGA). These materials have good biocompatibility and good tolerance and have been biodegraded by the hydrolysis of their ester linkages. [66,67]. This feature results in the main advantage of these materials, i.e., bioresorption, which means that these products do not require additional surgery to remove. 

### 3.6. Materials for Osteosynthesis

Plate osteosyntheses allow three dimensional reconstructions of complex face fractures and the skull base. The general standard treatment uses a titanium plate system because of its resistance to corrosion, strength, ease of handling, lack of dimensional changes, mechanical properties closest to the bone compared with the other metallic bioinert biomaterials, minimal scatter on computed tomography (CT) scanning, and compatibility with radiography and magnetic resonance imaging [68,69]. Titanium screws marketed in the internal fixation systems are commonly produced from a titanium alloy, Ti–6Al–4V alloy, which is the most widely used alloy. On the other hand, osteosynthesis plates are generally produced from CP-Ti (commercially pure titanium, usually grade 2) [70,71]. These materials have some disadvantages, such as poor resistance to wear, which results in the deposition of friction in the surrounding tissue, infections, and sensitivity perturbations [72]. The other issue is the secondary surgery needed for implant removal in 5–40% of cases [68], because of its translocation thermal sensitivity, interference with diagnostic imaging, osteopenia of cortical bone induced by stress, and corrosion [73,74]. Moreover, titanium particles, as products of wear, have been found in scar tissue covering these plates, as well as in locoregional lymph nodes [68]. Therefore, in some cases, titanium osteofixation implant materials should be removed after fulfilling their functions. Pinto et al. do not see the need for routine removal of these osteosynthesis implants after installation, except when there is a clinical indication that this should be done. There is, however, no consensus in the oral and maxillofacial surgery literature regarding the removal of bone plate in asymptomatic cases [71]. To minimize the above limitations, biodegradable bone fixation materials based on polyhydroxy acids (polyglycolide acid (PGA), polylactide (PLLA and PDLA)) have been developed [72,75,76,77,78,79] (Table 1).

The idea of biodegradable plates may have emerged from absorbable sutures [75]. The use of biodegradable materials to stabilize fractured facial skeletons was first reported in 1971 [74]. Since then, resorbable polymeric plates and screws have been used widely in pediatric patients with maxillofacial traumas, so as to reduce any interference with craniofacial growth in children [69,76]. The biodegradation process depends on many factors, such as: contact with body fluids, temperature, motion, molecular weight, the crystal form and geometry of the material, and the nature of the tissue where the implant is implanted. The ideal biodegradable osteofixation material provides appropriate strength while degrading in a predictable fashion throughout the healing process without causing adverse reactions. Recently, the concept has changed from simply “resorbable materials” to “bioabsorbable materials”, which means the materials have the characteristics of biodegradation plus stimulation of osteoconduction [75]. Unfortunately, they are weaker than conventional titanium plates and can provoke an inflammatory, bacterial foreign-body reaction [1,72,80]. However, Cural et al. found that resorbable (PDLLA) and titanium plates and screws did not differ in terms of biomechanical behaviors after stabilization of the fracture of the mandible angle [77], but the thickness of conventional bioresorbable plates is, on average, two to three times that of metal plates of comparable flexural strength [69]. Larger and thicker plates can lead to greater patient discomfort, as they may be palpable through the skin [78]. Furthermore, the thickness of the plates causes limitations in use—this can be a factor that influences the complication of plate exposure and wound dehiscence, especially in regions with very thin oral mucosa.

The mechanical properties of bioresorbable materials are close to that of the human bone, thereby preventing stress-shielding atrophy and weakening of the fixed bone caused by rigid metallic fixation [79]. Sukegawa et al. did not observe a border between the bone and u-HA/PLLA screws during their histological examination, indicating that the material directly bonded with the human bone [80]. Poly-L/D lactide plates and u-HA/PLLA composite plates are easily bendable with fingers at room temperature, combining wave-forms with angles and torsion, and can be maintained in the desired position without heating if slower bending and less force are applied [75]. However, long-term stability and relapse frequency in bioabsorbable osteofixation are still insufficiently studied, especially in cases concerning segmental movements of great magnitude or segmental movements to a position where bony resistance exists.

In contrast to metallic osteosynthesis, bioresorbable implants cannot be sterilized in the operating room through autoclaving. Manufacturers thus use either γ-irradiation or ethylene oxide gas (EtO) for sterilization of implants [81].

Magnesium has also been highlighted as a new material to replace polymer-based osteofixation material in maxillofacial bone surgery. The use of magnesium for bone implants was first described by Lambotte in 1932 [82]. The rapid corrosion of Mg and Mg alloys is a significant limitation regarding the use of these materials. Magnesium alloys possess good mechanical stability, which provides total degradability, but their biocompatibility is still questionable [81]. There is only one case report using Mg-based osteofixative materials in the maxillofacial area in humans [83]. Further research will be necessary to eliminate potential risk and to exclude the risk of non-biocompatibility.

### 3.7. Bone Substitution Materials

Bone is the second most transplanted tissue after blood [84]. Each bone defect within the maxillofacial skeleton resulting from trauma, disease (i.e., tumors, cysts), or congenital malformation is a significant health problem. Biomaterials used as bone grafts must meet specific requirements to achieve new, healthy, well-vascularized bone tissue formation. Autologous, allogenic, alloplastic, or xenogenic materials are used in bone regeneration [85]. Although autologous bone is still the gold-standard graft material and is not a biomaterial per se, other grafts are used very often (alone or in combination) [86]. A large variability exists between the bone-forming capabilities of various bone grafts, and the osteoinductive potential remains one of the key features to improve the integration of implanted bone grafts. For the regeneration of small osseous defects, bone-substitute biomaterials covered by a membrane are commonly used.

DBBM (deproteinized bovine bone mineral) is the biomaterial with the most documentation in the scientific literature for bone grafting [87]. Deproteinized bone matrix of cortical or cancellous xenogenic bone used as a bone graft material shows biocompatibility, provides a supportive osteoconductive structure, and releases calcium and phosphate ions, thus stimulating osteogenesis. However, when the proteins in its structure are not fully eliminated prior to use, it may provoke foreign body reactions. In addition, there is a potential for cross-infection [88,89]. Although the authors have not found a case of infection from xenotransplantation in maxillofacial surgery, despite them having been reported, there is a potential risk and certain precautions must be taken. 

Commercially available xenogenic osteoconductive biomaterials are made of bovine bone (e.g., Bio-Oss^®^, Gen-Ox^®^, Cerabone), porcine bone (e.g., Gen-Os), or horse bone (e.g., Bio-Gen). The deproteinization processes for xenogenic grafts can be performed by chemical or heat treatments. Uklejowski et al. showed that the thermal deproteinization process leads to numerous cracks on the surface of the trabeculae of cancellous bone but is much shorter, while bone specimens after the deproteinization process with the chemical agents are generally smooth [90]. According to their study, the most complete and most effective chemical deproteinization process is obtained when using 7 wt% H_2_O_2_ solution —bone specimens are deproteinized by 90% after 14 days of process. Due to the mechano-structural properties and effectiveness, the chemical deproteinization processes are more suitable for bone tissue replacements [90]. 

Synthetic materials are not as widely accepted as the allograft materials, despite their obvious benefits; they still lack a significant amount of documented clinical studies supporting their effectiveness. The most investigated calcium phosphate (CaP) bone graft substitutes are hydroxyapatite (HA), B-tricalcium phosphate (B-TCP), and their combination, also called biphasic calcium phosphate (BCF) [71,90]. Their bioactivity and degradation time can be controlled by changing their chemical compositions and sintering temperatures. When compared to synthetic polymers, synthetic bioceramics are superior for bone repairs due to their improved biocompatibility, bioactivity, and strength [71,91]. Yahav et al. show that biphasic calcium sulfate sets hard, acting like a “bone cement”, no membrane is required, and primary closure is not mandatory. The material has a complete conversion to bone over a period of four to six months. They achieved similar clinical results as with other grafting products [92]. Miron et al., in a study of the osteoinductive potential of bone grafting material, showed that the xenograft (DBBM) has no potential to form ectopic bone formations, but BCP (biphasic calcium phosphate fabricated from a 10:90 ratio of hydroxyapatite and β-tricalcium phosphate) was able to stimulate ectopic bone formation [93]. Donos et al. obtained similar results in relation to DBBM [94]. Guillaume obtained satisfactory results for bone regeneration with B-TCP for pre-implant surgery, sinus floor elevation, and lateralization of the inferior alveolar nerve (IAN) [95]. 

Besides the “traditional” use for osseous defect repair, a variety of innovative applications are emerging; for instance, recent studies have interestingly highlighted the suitability of bioactive glasses and glass–ceramics for wound healing applications and soft tissue engineering. 

**Figure 7:**
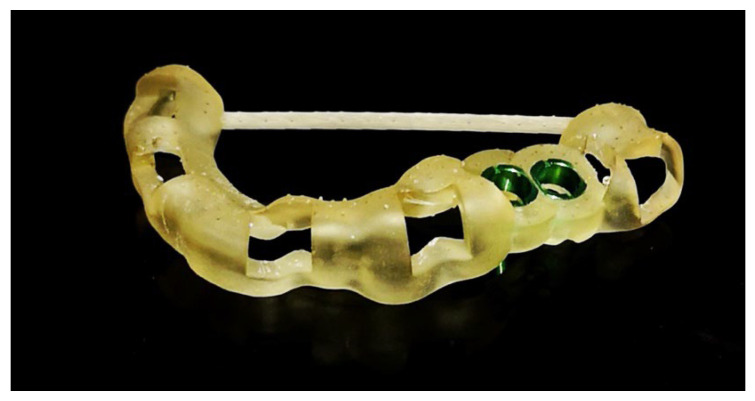
Surgical templates printed using a 3D printer personalized to the patient’s anatomical features, fitted on CT scan and oral scanner. Used to facilitate and speed up the surgical procedure of dental implants. Material from the authors’ collection.

Even though the ideal properties of bone grafts were defined in the literature three decades ago, the market still has no ideal biomaterial which has all of these properties [71]. In a consensus report of Group 2 of the 15 h European Workshop on Periodontology on Bone Regeneration we can read that the future of craniomaxillofacial bone regeneration will probably entail the manufacturing of personalized biomaterial from 3D digital data obtained from patients [90]. Manufacturing customized scaffolds or bones with 3D printing that will perfectly fit to the bone defect shape is a dream of many scientists and surgeons. So far, surgical templates printed using a 3D printer have been increasingly used to facilitate and speed up the surgical procedure (Figure 7). 

Bone regeneration techniques need resorbable or non-resorbable membranes as well. The barrier membranes prevent the invasion of surrounding soft tissue, provide stability to the bone graft, prevent soft tissue from collapsing into the defect, accumulate growth factors, and permit osteogenic cells to repopulate bone defects. [96,97,98]. At present, resorbable materials of xenogeneic origin, such as collagen, are the most commonly used option in guided bone regeneration [98]. However, PTFE membranes also have many uses. Compared with biodegradable membranes, they have a superior space-making capability, mainly when they have titanium reinforcement, which makes them the ideal membranes for vertical bone regeneration [90]. Garcia et al. systematically reviewed the available literature to ascertain the clinical outcomes of two different resorbable collagen membranes and concluded that GBR procedures, through resorbable collagen membranes, achieve volumetric bone gains with no statistical significance between the cross-link and the non-cross-link membranes. However, in terms of biocompatibility, tissue integration, and postoperative complications, the results suggest that non-cross-link membranes present better results [99]. Sbricoli et al. reached a similar conclusion, i.e., that collagen membranes show advantageous biological and clinical features compared to both non-resorbable and other resorbable membranes, but they are not free from possible complications [100].

Martin-Thomé et al. undertook a case series study of a bi-layered synthetic resorbable PLGA membrane (Tisseos^®^, Biomedical Tissues SAS, 129 Nantes, France). This membrane is made of poly-D,L-lactic/glycolic acid 85/15 (PLGA) and completely degrades by hydrolysis after 4–6 months without signs of inflammation and has a bi-layered structure with a dense film to prevent gingival epithelial cell invasion and a micro-fibrous layer to support osteogenic cells and bone healing. Re-epithelialisation and normal wound closure were observed in patients, where the membrane was exposed after surgery [101].

### 3.8. Voice Prosthesis

**Figure 8:**
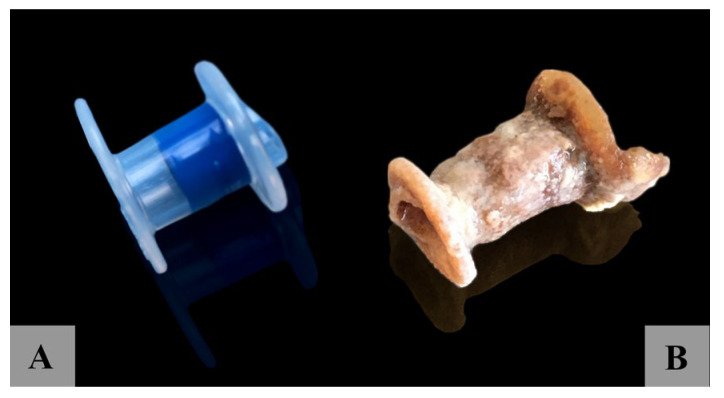
Figure presents Provox voice prostheses. Panel (**A**) shows a completely new prosthesis. Panel (**B**) shows the voice prosthesis after 26 months of use. Its surface is covered with microbial biofilm. Material from the authors’ collection.

The most common and effective method of voice rehabilitation among post-laryngectomy patients is a tracheoesophageal puncture with voice prosthesis (VP) implantation [102]. The most serious disadvantage of silicone-polymer-based voice prosthesis devices is their colonization and damage by fungi and bacterial biofilm [9,10] (Figure 8). The most common yeasts isolated from VPs’ biofilms are *Candida* spp., which forms a tridimensional network leading to device malfunction [103]. There are few voice prostheses manufacturers in the market, but, beyond the prosthesis shape and insertion procedure technique, the polymer material is generally still the same as it has been for years. The new polymeric material should be improved to prevent or slow down the VP degradation process. A modification that will result in antimicrobial properties would be highly desirable. To achieve such a goal, commonly used polymers should be modified with antimicrobial nanosystems or chemical compounds [104,105,106,107,108] that might reduce the ability of microorganisms to adhere to and develop biofilms on the prosthesis’ surface. Another approach is to find materials that will have relatively better physical and/or antimicrobial features and/or bacterial and fungal anti-attachment properties. Among popular polymers, some authors selected nine because of their relatively good features, such as the polymers’ costs, chemistries, and toxicities. They have found that AODMBA [(R) α acryloyloxy β,β dimethyl γ butyrolactone] was demonstrated to be 3D printable and exhibited strong anti attachment properties, which were retained in its AODMBA printed forms. These tests showed that anti attachment by AODMBA is just as effective against the drug-resistant isolates as against a standard *C. albicans* strain [109]. Further investigations should be performed in this area. Looking for methods that will prevent the initial adherence of hyphae (an essential step in biofilm formation) should be considered essential in the development of future VP material.

### 3.9. Tissue Engineering

Tissue engineering (TE) has the potential for reconstruction with autologous tissue that is not limited by availability of patient donor-site tissue. TE is applicable in otolaryngology in nose, external ear, laryngo-tracheal, and facial skeleton reconstruction [110]. Otolaryngology has a symbolic association with TE because of the memorable picture of the Vacanti mouse bearing a human ear on its back from 1996 [111]. Nowadays, for nasal reconstruction, tissue-engineered cartilaginous constructs are alternatives for synthetic or allogenic materials. The method is based on the implantation of biodegradable collagen scaffolds with seeded chondrocytes and progenitor cells instead of nasal cartilages. The literature reports cases of reconstructions of the two-layer alar lobule or the nasal dorsum in patients after tumor resections or cleft lip–nose deformities. They have achieved good aesthetic and functional outcomes using autologous nasal septal chondrocytes seeded on utilized collagen membranes or scaffolds [112,113]. As with the nasal TE, the otologic appliance has focused on auricular reconstructions. Investigators expanded harvested microtia chondrocytes, seeded these on a 3D-printed biodegradable scaffold, and cultured the construct in vitro. They reported satisfactory post-implantation aesthetic outcomes [114]. On the other hand, bacterial nanocellulose (BNC), which is non-degradable biocompatible material that promotes chondrocyte adhesion and proliferation, also exists. Nimeskern et al. presented BNC as having the capability to reach mechanical properties of relevance for ear cartilage replacement; it can be produced in patient-specific ear shapes [115]. 

There are also investigations into the regeneration of inner- and middle-ear structures. Chitosan patches (E-CPs) that release epidermal growth factor (EGF) as a patch therapy to replace surgical methods of the perforated tympanic membrane reconstructions have also been developed [116]. The inner-ear treatment by TE is focused on in vitro models of decellularized cochleae and the establishment of pluripotent stem cell lines with the goal of generating functional inner-ear hair cells [117]. Regenerative medicine for laryngotracheal replacements has, in recent years, focused on investigations concerning ideal scaffold materials for tracheal reconstruction [110]. Commonly used scaffold materials include decellularized tissue, poly-lactic-co-glycolic acid (PLGA), poly-ε-caprolactone (PCL), polyethylene terephthalate (PET), and polyurethane (PU). There is still no answer as to which material is optimal for this procedure [118,119,120,121]. Simultaneously, investigators are examining the ideal cellular source for graft seeding. Moreover, some in vivo animal model studies with stem cell-seeded constructs were performed with positive outcomes in restoring larynx phonation function [122,123].

## 4. Improving the Safety of Biomaterials by Preventing Their Microbial Colonization and Host Immune Response

Despite the extremely important role of newly formed biomaterials in improving the health and quality of the lives of patients with dysfunctions within the head and neck region, not all safety issues related to their functions within the patients’ bodies have been resolved. One of the greatest risks associated with placing an implant within the living tissue of a patient is related to the colonization of the material by opportunistic/pathogenic microorganisms and the formation of a bacterial/fungal biofilm on the implant’s surface. This is an important issue, because the presence of microorganisms on the surface of the implant not only changes its mechanical properties, accelerates its wear [10], and increases the risk of explanation [124], but also, above all, can be a source of life-threatening infections leading to the development of sepsis [125,126]. Under the conditions of the human body, excessive colonization of tissue surfaces by pathogenic microorganisms is limited due to specific, mucilaginous barriers, the presence of natural microflora, and the synthesis of a number of endogenous substances characterized by antibacterial and immunomodulatory activity. In the case of implanted biomaterials, such protection does not occur, which forces the search for protective methods limiting the formation of biofilm on their surface to take place. In other words, the restoration of the live tissue’s ability to prevent microbial colonization should be taken into consideration within the design of materials for implant production. 

The group of methods limiting microorganism growth and hampering microbial adherence includes the fabrication of materials with anti-fouling features [127], covering the surface of implants with anti-adhesive substances [128], surface charge modifications [129], coating with antibiotics and antimicrobial peptides [130], or the use of nanoparticles as antimicrobial covering. Particularly, the use of nanotechnological methods has recently attracted more interest due to their lower potential to induce microbial drug resistance and both potent, broad-spectrum antimicrobial activity and immunomodulatory features. The above-mentioned methods and their applications are presented in Table 2.

### 4.1. Methods of Biomaterial Modifications to Increase Their Antimicrobial Properties

Antibacterial coating. There are three main methods for fixing antibacterial agents on the material surface: (i) covalent grafting, (ii) material blending, and (iii) layer-by-layer (LBL) assembly. Covalent grafting is more stable than other methods, such as non-covalent bonding (electrostatic attraction and hydrogen bonding). There are some very detailed publications describing two categories of covalent grafting—‘grafting to’ and ‘grafting from’ [136,137,138,139]. Moreover, the technology of photo-initiation of some monomers or polymers by UV also exists. This technique can be very efficient to prevent the formation of bacterial biofilms on medical devices [140]. 

The LBL assembly is considered to be a universal technique for making antimicrobial coatings on medical devices. It relies on the adsorption of electrolytes or complementary compounds on the substrate surface [141]. The applications of this method are very wide-ranging, there are some works that report the modification of different materials by LBL assembly as: polyacrylic acid (PAA, as the polyanion) and polyetherimide (PEI, as the polycation) used to obtain a multilayer PAA/PEI assembled film [142], chitosan and heparin [143], chitosan and collagen [144], polydimethylaminoethyl methacrylate (polycation) and cellobiose dehydrogenase (CDH, polyanion), and polybenzenesulfonic salt (polyanion) [145]. 

Material blending is a method of mixing different types of polymers. The final antimicrobial effects depend on the features of the used substrates and their proportions. This technology was used in polyhexamethyleneguanidine dodecylbenzenesulfonate (PHMG- DBS)-coated tracheal intubation tubes (good antimicrobial outcomes were reported) [146]. Indeed, a polyurethane (PU) catheter modified with poly(diallyldimethylammonium chloride) (pDADMAC) demonstrated good bactericidal features [147].

Antibiotic delivery systems. In some cases, polymers could serve as an antibiotic-controlled release systems. Rossi et al. described their method of incubation of poly(hydroxybutyric-co-hydroxyvalerate) (PHBV) in a solution of chloroform and gentamicin in a shaking water bath at 55 °C for 24 h. The authors reported a good profile of drug release and good bactericidal effect [148]. Another study reported that the release of antibiotics from different material formats of silk (films, microspheres, hydrogels, coatings) and biodegradable chitosan had good functional profiles and the potential to achieve the needed local concentrations while also minimizing systemic exposure [149,150]. 

Polymers with nanoparticles. Silver nanoparticles alone have a proven bactericidal effect in the treatment of local infections. The mechanism of their action is based on the ability to damage the bacterial cell membrane. There are some studies that report the method of silver nanoparticles’ incorporation into polymer materials to achieve antifungal and bactericidal features. Polymer systems containing silver nanoparticles can be synthesized in situ using the polymer matrix as a reaction medium or ex situ when silver nanoparticles are incorporated into the polymeric matrix already synthetized [151,152]. The latest studies report the use of silver nanoparticles modified with zwitterionic poly (carboxybetaine-co-dopamine methacrylamide) copolymer (PCBDA@AgNPs), which was firmly fixed onto soft contact lenses through the mussel-inspired surface chemistry [153] or hybrid nanocoatings [154]. Ye et al. described the method of connection between antimicrobial peptides, GL13K and AgNP, to create a hybrid nanocoating on a Ti implant. Due to the combined application of these two antimicrobial agents with different antimicrobial mechanisms, they achieved more potent synergistic effects. 

### 4.2. Selected Biomaterials with Antimicrobial Modifications for Use in Otorhinolaryngology

In one of the more interesting studies, Duda et al. presented the possibility of coating Bioverit^®^ II middle ear prostheses using silver containing silica. In their study, rabbits were implanted with silver-functionalized middle-ear prostheses, and this group was compared with pure silica coatings and 1% silver sulfadiazine cream applied on a silica coating. The authors demonstrated the clinical justification of the study, and the usefulness of the developed implants for reconstructive middle-ear surgery and reduced fibrosis was observed. Nevertheless, some signs of acute toxicity of the silver coatings on the mucosal ear tissue (particularly single necrotic and apoptotic cells) were observed, which prompted further studies into the safety of such an approach [155]. Another problem is the formation of bacterial biofilm on medical devices, such as cochlear implants (CI), that can lead to chronic infections. In response to this, a vancomycin-releasing PCL/polyethylene oxide (PEO) nanofiber mat was proposed to prevent MRSA biofilm formation on the surface of ossicular prostheses and in the environment of otitis media, due to effective delivery of vancomycin for prolonged time [156]. 

Kirchhoff et al. found in vitro that bioactive glass (BAG of type S53P4) consisting of silicon dioxide, sodium oxide, calcium oxide, and phosphorus pentoxide induces significant changes in the biofilm morphology of the most common bacterial strains responsible for biofilm-related implant infections. Its antibacterial activity was tested with three types of materials commonly used in cochlear implants: silicone, titanium, and platinum. In each case, significant alterations in biofilm morphology could be detected via SEM. Höing et al. also found that bioactive glass S53P4 can reduce biofilm formation on CI materials in vitro [131].

The formation of fungal biofilm can be seen as a separate problem in the implementation of medical devices. Filastatin inhibits the adhesion of multiple pathogenic *Candida* species. Vargas-Blanco et al. report that treatment with Filastatin significantly inhibited the ability of *C. albicans* to adhere to bioactive glass (by 99.06%), silicone (by 77.27%), and dental resin (by 60.43%) in vivo.

**Figure 9:**
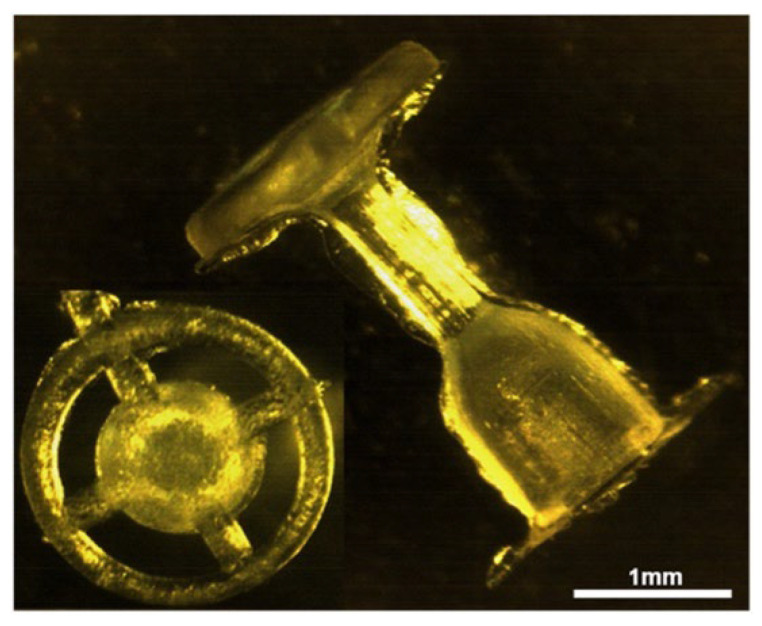
Middle-ear prosthesis for ossicular reconstruction made of ABS polymer. Adapted from an open-access source: [158].

The first middle-ear implant that has the bactericidal activity and hearing improvement confirmed in the clinical trial conducted by Ziąbka and Malec [157] (Figure 9).

Recent experimental ossicular chain implants made of ABS polymer material (poly)acrylonitrile butadiene styrene (INEOS SyrolutionEurope GmbH, Frankfurt, Germany) and modified with silver nanoparticles, AgNPs 45T, have shown promising antimicrobial efficacy [57,159,160]. Such activity is regarded as being the same as biocompatibility or physical properties. 

Silver nanoparticles, used to provide antimicrobial activity, were also used as coatings for nasal tampons [134]. Research carried out using rats to test such tampons when applied for 48 h demonstrated that silver nanoparticle-embedded tampons were far more efficient in limiting *Haemophilus parainfluenzae* colonization when compared to silicone- or PEG-based nasal splints. A decreased inflammatory response was also noted, which suggest the utility of AgNPs in the prevention of secondary infections [134]. A nanotechnology approach was also used for the preparation of ciprofloxacin and azithromycin nanoparticle suspension for the coating of sinus stents with anti-*Pseudomonas aeruginosa* properties [133].

In the context of bone substitution materials, nanohydroxyapatite can be loaded with chlorhexidine digluconate using electrostatic interactions between the cationic group of CHX and the phosphate group of nanoHA in order to prevent surface bacterial accumulation [159]. Nevertheless, in the case of bone infections, poor bone/plasma ratios of parenterally administrated antibiotics, along with the ability of bacteria to form biofilm inside the bone, considerably hampers the fighting of bacterial infections. To increase the amount and efficiency of antibiotics against bone infection-causing pathogens, Parent et al. proposed the incorporation of vancomycin in tri-dimensional hydroxyapatite-based scaffolds for local, prophylactic delivery of antibiotics [132]. Similarly, Weng et al. demonstrated recently that a local three-dimensional scaffold strategy strongly contributes to the improvement of anti-MRSA therapies, as a result of coating the bone biomaterial with a significant amount of silver nanoparticles [160].

Another promising approach for decreasing bacterial adherence to the implants is surface–charge modification. Kao et al. found in in vivo models, that charge modification decreased the colonization by *P. aeruginosa* of titanium screw implants. 

Another method to improve the function of biomaterials and protect implants from microbial exposure is to modulate the inflammatory response of the host. Nevertheless, this effect should be balanced between a beneficial, antimicrobial effect and a redundant, toxic effect that damages tissues and increases the risk of inflammation. 

Although the number of studies examining the immunomodulatory activity of biomaterials coatings has decreased when compared to studies examining antimicrobial ones, some interesting studies have been undertaken recently. In one of the newer studies, lithium niobate nanoparticles were proven to exert an immunomodulatory effect, possess the ability to stimulate beta-defensin in epithelial cells, and shown to be effective against *Pseudomonas aeruginosa* bacteria, which supports their utility for inner-ear-device fabrication [135]. In another study, magnetic hexagonal ferrite nanoparticles were incorporated into bone hydroxyapatite/chitosan scaffolds to recruit endogenous stem cells [161]. The promising results were also obtained using a mouse soft-tissue implant-associated infection model, where anti-biofilm and the immunomodulatory activities of zinc oxide nanoparticle (ZnO NPs) were investigated. According to Wang et al., this effect was achieved by a combination of factors, including (i) the antimicrobial efficiency of ZnO NPs, (ii) the nanoparticle-mediated induction of inflammatory cytokines release, and (iii) promoting phagocytosis [162]. Although the results of these studies are not application specific, they can certainly be thought of in terms of their subsequent use in the production of implants in otorhinolaryngology. Zinc-incorporated titanium oxide nanotubes were also noted to accelerate bone formation when fabricated on titanium implant materials [163].

The above research confirms that the development of implantable biomaterials with an appropriate safety–effectiveness ratio is extremely important and worthy of further research.

## 5. Conclusions

In general, the most widely used materials in the field of otorhinolaryngology are polymer-based synthetics. They have the potential to be an ideal material in this case, if it is possible to achieve modification of their properties, such as the ability to prevent the growth of microorganisms. As we currently face the challenge of an increasing number of infections caused by antibiotic-resistant strains of bacteria, there is an urgent need to find a new class of antibacterial agents. AMPs, their synthetic mimics, and nanosystems containing metallic nanoparticles have a promising potential as a more efficient alternative to conventional antibiotics, due to their microbiocidal properties that cover large spectrum of microorganisms, as well as their low ability to induce drug resistance. There is no doubt that the development of these molecules for the antibacterial modification of materials used in medical devices in otorhinolaryngology will be beneficial. At the same time, we should look for new materials that will have better biocompatibility and mechanical properties. The ability to customize the shapes of this biomaterial with 3D printers will also be of benefit to patients in need.

## Figures and Tables

**Table 1:** Characteristics of bioresorbable materials and commercially available systems for maxillofacial osteosynthesis.

Material	PGA Polyglycolic Acid	PLA Polylactic Acid		Copolymers of PGA, PLLA, PDLA	uHA/PLLA Composites of Unsintered Hydroxyapatite and Poly-L-Lactide
		PLLA Poly-L-Lactide	PDLA Poly-D-Lactide		
		1st Generation		2nd Generation	3rd Generation
**Application**	High molecular weight; highly crystalline; rapidly degradable; radiotransparency; first bioresorbable polymer clinically used.	High molecular weight due to its crystallinity and hydrophobicity; resistant to hydrolysis; radiotransparency.	High molecular weight; lower crystallinity; less resistant to hydrolysis; highly biocompatible compared to PLLA radiotransparency.	Their properties can be controlled by varying the ratio of glycolide to lactide for different compositions. Radiotransparency.	Contains 30–40% weight fractions of raw hydroxyapatite, neither calcined nor sintered material. Osteoconductive capacity (can be complete replacement by bone tissue); radiopacity.
	Early loss of mechanical strength after 4–7 weeks, clearance time is 6–12 months [79]	Total resorption is over 3.5 years in vivo, in vitro about 2 years [79]	-	Resorption time of 12–18 months. In general, a higher glycolide content leads to a faster rate of degradation.	The PLLA matrix is completely absent from the composites after 4 years and almost all u-HA particles are replaced after 5.5 years [77]
	Pure PGA, due to its durability, which is insufficient to allow for complete bone healing, has rather minimal usefulness in maxillofacial surgery [68]. Biofix^®^ SR-PGA (self-reinforced PGA).	GrandFix^®^ FixSorb-MX^®^	There is no study using pure PDLA for osteofixation in the maxillofacial surgery.	SonicWeld Rx^®^ (PDDLA 100%) LactoSorb^®^ (PLLA (82%) + PGA (18%)) RapidSorb^®^ (PLLA 85% + PGA 15%) Delta^®^ PLLA (85%) + PGA (10%) + PDLA (5%) PolyMax^®^ (PLLA70% + PLDLA (30%)	Osteotrans MX^®^ (plate: PLLA 60 wt% + u-HA 40 wt%; screw: PLLA (70 wt%) + u-HA (30 wt%))

**Table 2:** Various methods to synthetize materials with antimicrobial properties and their uses.

Method of Material Antimicrobial Functionalization	Application
anti-fouling	cochlear implants [127,131]
anti-adhesive	dental resin and polydi-methylsiloxane elastomer (PDMS) [128]
surface–charge modification	titanium micro-screws [129]
coating with antibiotics	bone implants [132], tympanostomy tubes [41], paranasal sinus stents [133],
coating with antimicrobial agent such as nanoparticles	nasal mucosa splints [134], inner ear implants [135], middle ear implants [54], bone tissue scaffolds [86,90,91]

## Data Availability

Publicly available datasets were analyzed in this study. This data can be found in PubMed.

## References

[1] Bergman C.P., Aisha S. (2013). Dental Ceramics: Microstructure, Properties and Degradation.

[2] Sternberg K. (2009). Current requirements for polymeric biomaterials in otolaryngology. GMS Curr. Top. Otorhinolaryngol. Head Neck Surg..

[3] Jones J.R., Hench L.L. (2001). Biomedical materials for new millennium: Perspective on the future. Mater. Sci. Technol..

[4] Hermawan H., Mantovani D. (2009). Degradable metallic biomaterials: The concept, current developments and future directions. Minerva Biotecnol..

[5] Bohner M. (2010). Resorbable biomaterials as bone graft substitutes. Mater. Today.

[6] Mathur A.B., Collier T.O., Kao W.J., Wiggins M., Schubert M.A., Hiltner A., Anderson J.M. (1997). In vivo biocompatibility and biostability of modified polyurethanes. J. Biomed. Mater. Res..

[7] Heumann S., Eberl A., Pobeheim H., Liebminger S., Fischer-Colbrie G., Almansa E., Cavaco-Paulo A., Gubitz G.M. (2006). New model substrates for enzymes hydrolysing polyethyleneterephthalate and polyamide fibres. J. Biochem. Biophys. Methods.

[8] King R.N., Lyman D.J. (1975). Polymers in contact with the body. Environ. Health Perspect..

[9] Galli J., Calo L., Meucci D., Giuliani M., Lucidi D., Paludetti G., Torelli R., Sanguinetti M., Parrilla C. (2018). Biofilm in voice prosthesis: A prospective cohort study and laboratory tests using sonication and SEM analysis. Clin. Otolaryngol..

[10] Spałek J., Deptuła P., Cieśluk M., Strzelecka A., Łysik D., Mystkowska J., Daniluk T., Król G., Góźdź S., Bucki R. (2020). Biofilm Growth Causes Damage to Silicone Voice Prostheses in Patients after Surgical Treatment of Locally Advanced Laryngeal Cancer. Pathogens.

[11] Lendlein A. (1999). Polymere als Implantatwerkstoffe. Chem. Unserer Zeit.

[12] Unverdorben M., Spielberger A., Schywalsky M., Labahn D., Hartwig S., Schneider M., Lootz D., Behrend D., Schmitz K., Degenhardt R. (2002). A polyhydroxybutyrate biodegradable stent: Preliminary experience in the rabbit. Cardiovasc. Interv. Radiol..

[13] Sodian R., Hoerstrup S.P., Sperling J.S., Martin D.P., Daebritz S., Mayer J.E., Vacanti J.P. (2000). Evaluation of biodegradable, three-dimensional matrices for tissue engineering of heart valves. ASAIO J..

[14] Ray S., Kalia V.C. (2017). Biomedical Applications of Polyhydroxyalkanoates. Indian J. Microbiol..

[15] Parikh A., Anand U., Ugwu M.C., Feridooni T., Massoud E., Agu R.U. (2014). Drug-eluting nasal implants: Formulation, characterization, clinical applications and challenges. Pharmaceutics.

[16] Blasi P. (2019). Poly(lactic acid)/poly(lactic-co-glycolic acid)-based microparticles: An overview. J. Pharm. Investig..

[17] Elmowafy E.M., Tiboni M., Soliman M.E. (2019). Biocompatibility, biodegradation and biomedical applications of poly(lactic acid)/poly(lactic-co-glycolic acid) micro and nanoparticles. J. Pharm. Investig..

[18] Schoubben A., Ricci M., Giovagnoli S. (2019). Meeting the unmet: From traditional to cutting-edge techniques for poly lactide and poly lactide-co-glycolide microparticle manufacturing. J. Pharm. Investig..

[19] Zhong H., Chan G., Hu Y., Hu H., Ouyang D. (2018). A Comprehensive Map of FDA-Approved Pharmaceutical Products. Pharmaceutics.

[20] Long M., Rack H.J. (1998). Titanium alloys in total joint replacement—A materials science perspective. Biomaterials.

[21] Yazdimamaghani M., Razavi M., Vashaee D., Moharamzadeh K., Boccaccini A.R., Tayebi L. (2017). Porous magnesium-based scaffolds for tissue engineering. Mater. Sci. Eng. C Mater. Biol. Appl..

[22] Marti A. (2000). Cobalt-base alloys used in bone surgery. Injury.

[23] Ortiz A.J., Fernandez E., Vicente A., Calvo J.L., Ortiz C. (2011). Metallic ions released from stainless steel, nickel-free, and titanium orthodontic alloys: Toxicity and DNA damage. Am. J. Orthod. Dentofac. Orthop..

[24] Gomes C.C., Moreira L.M., Santos V.J., Ramos A.S., Lyon J.P., Soares C.P., Santos F.V. (2011). Assessment of the genetic risks of a metallic alloy used in medical implants. Genet. Mol. Biol..

[25] Zhao D., Witte F., Lu F., Wang J., Li J., Qin L. (2017). Current status on clinical applications of magnesium-based orthopaedic implants: A review from clinical translational perspective. Biomaterials.

[26] Lourenço M.L., Cardoso G.C., dos Santos Jorge Sousa K., Donato T.A.G., Pontes F.M.L., Grandini C.R. (2020). Development of novel Ti-Mo-Mn alloys for biomedical applications. Sci. Rep..

[27] Jakubowicz J. (2020). Special Issue: Ti-Based Biomaterials: Synthesis, Properties and Applications. Materials.

[28] Yang K., Ren Y. (2010). Nickel-free austenitic stainless steels for medical applications. Sci. Technol. Adv. Mater..

[29] Aldini N.N., Fini M., Giavaresi G., Torricelli P., Martini L., Giardino R., Ravaglioli A., Krajewski A., Mazzocchi M., Dubini B. (2002). Improvement in zirconia osseointegration by means of a biological glass coating: An in vitro and in vivo investigation. J. Biomed. Mater. Res..

[30] Saenz A., Brostow W., Rivera-Muñoz E. (1999). Ceramic biomaterials: An introductory overview. J. Mater. Educ..

[31] Cranin A.N., Schnitman P.A., Rabkin S.M., Onesto E.J. (1975). Alumina and zirconia coated vitallium oral endosteal implants in beagles. J. Biomed. Mater. Res..

[32] Depprich R., Zipprich H., Ommerborn M., Naujoks C., Wiesmann H.P., Kiattavorncharoen S., Lauer H.C., Meyer U., Kübler N.R., Handschel J. (2008). Osseointegration of zirconia implants compared with titanium: An in vivo study. Head Face Med..

[33] Scholz M.S., Blanchfield J.P., Bloom L.D., Coburn B.H., Elkington M., Fuller J.D., Gilbert M.E., Muflahi S.A., Pernice M.F., Rae S.I. (2011). The use of composite materials in modern orthopaedic medicine and prosthetic devices: A review. Compos. Sci. Technol..

[34] Lenarz T., Lesinski-Schiedat A., Weber B.P., Frohne C., Buchner A., Battmer R.D., Parker J., von Wallenberg E. (1999). The Nucleus Double Array Cochlear Implant: A new concept in obliterated cochlea. Laryngorhinootologie.

[35] Scheper V., Hessler R., Hutten M., Wilk M., Jolly C., Lenarz T., Paasche G. (2017). Local inner ear application of dexamethasone in cochlear implant models is safe for auditory neurons and increases the neuroprotective effect of chronic electrical stimulation. PLoS ONE.

[36] Wilk M., Hessler R., Mugridge K., Jolly C., Fehr M., Lenarz T., Scheper V. (2016). Impedance Changes and Fibrous Tissue Growth after Cochlear Implantation Are Correlated and Can Be Reduced Using a Dexamethasone Eluting Electrode. PLoS ONE.

[37] Lehner E., Gundel D., Liebau A., Plontke S., Mader K. (2019). Intracochlear PLGA based implants for dexamethasone release: Challenges and solutions. Int. J. Pharm. X.

[38] Fredenberg S., Wahlgren M., Reslow M., Axelsson A. (2011). The mechanisms of drug release in poly(lactic-co-glycolic acid)-based drug delivery systems—A review. Int. J. Pharm..

[39] Plontke S.K., Glien A., Rahne T., Mader K., Salt A.N. (2014). Controlled release dexamethasone implants in the round window niche for salvage treatment of idiopathic sudden sensorineural hearing loss. Otol. Neurotol..

[40] Jang C.H., Park H., Cho Y.B., Choi C.H., Park I.Y. (2009). The use of piperacillin-tazobactam coated tympanostomy tubes against ciprofloxacin-resistant *Pseudomonas* biofilm formation: An in vitro study. Int. J. Pediatr. Otorhinolaryngol..

[41] Jang C.H., Park H., Cho Y.B., Choi C.H. (2010). Effect of vancomycin-coated tympanostomy tubes on methicillin-resistant *Staphylococcus aureus* biofilm formation: In vitro study. J. Laryngol. Otol..

[42] Saidi I.S., Biedlingmaier J.F., Whelan P. (1999). In vivo resistance to bacterial biofilm formation on tympanostomy tubes as a function of tube material. Otolaryngol. Head Neck Surg..

[43] Jang C.H., Cho Y.B., Choi C.H. (2012). Effect of ion-bombarded silicone tympanostomy tube on ciprofloxacin-resistant *Pseudomonas aeruginosa* biofilm formation. Int. J. Pediatr. Otorhinolaryngol..

[44] Joe H., Seo Y.J. (2018). A newly designed tympanostomy stent with TiO_2_ coating to reduce *Pseudomonas aeruginosa* biofilm formation. J. Biomater. Appl..

[45] de Abajo J., Sanhueza I., Giron L., Manrique M. (2013). Experience with the active middle ear implant in patients with moderate-to-severe mixed hearing loss: Indications and results. Otol. Neurotol..

[46] Stupp C.H., Stupp H.F., Grün D. (1996). Replacement of ear ossicles with titanium prostheses. Laryngorhinootologie.

[47] Maassen M.M., Löwenheim H., Pfister M., Herberhold S., Jorge J.R., Baumann I., Nüsser A., Zimmermann R., Brosch S., Zenner H.P. (2005). Surgical-handling properties of the titanium prosthesis in ossiculoplasty. Ear Nose Throat J..

[48] Gostian A.O., Kouame J.M., Bremke M., Ortmann M., Hüttenbrink K.B., Beutner D. (2016). Long term results of the titanium clip prosthesis. Eur. Arch. Otorhinolaryngol..

[49] Wongwiwat P., Boonma A., Lee Y.S., Narayan R.J. (2011). Bioceramics in ossicular replacement prostheses: A review. J. Long Term. Eff. Med. Implant..

[50] Shinohara T., Gyo K., Saiki T., Yanagihara N. (2000). Ossiculoplasty using hydroxyapatite prostheses: Long-term results. Clin. Otolaryngol. Allied Sci..

[51] Ocak E., Beton S., Meço C., Dursun G. (2015). Titanium versus Hydroxyapatite Prostheses: Comparison of Hearing and Anatomical Outcomes after Ossicular Chain Reconstruction. Turk. Arch. Otorhinolaryngol..

[52] Meijer A.G., Segenhout H.M., Albers F.W., van de Want H.J. (2002). Histopathology of biocompatible hydroxylapatite-polyethylene composite in ossiculoplasty. ORL J. Otorhinolaryngol. Relat. Spec..

[53] Ovsianikov A., Chichkov B., Adunka O., Pillsbury H., Doraiswamy A., Narayan R.J. (2007). Rapid prototyping of ossicular replacement prostheses. Appl. Surf. Sci..

[54] Ziabka M., Menaszek E., Tarasiuk J., Wronski S. (2018). Biocompatible Nanocomposite Implant with Silver Nanoparticles for Otology-In Vivo Evaluation. Nanomaterials.

[55] Krings J.G., Kallogjeri D., Wineland A., Nepple K.G., Piccirillo J.F., Getz A.E. (2014). Complications of primary and revision functional endoscopic sinus surgery for chronic rhinosinusitis. Laryngoscope.

[56] Okushi T., Yoshikawa M., Otori N., Matsuwaki Y., Asaka D., Nakayama T., Morimoto T., Moriyama H. (2012). Evaluation of symptoms and QOL with calcium alginate versus chitin-coated gauze for middle meatus packing after endoscopic sinus surgery. Auris Nasus Larynx.

[57] Verim A., Seneldir L., Naiboglu B., Karaca C.T., Kulekci S., Toros S.Z., Oysu C. (2014). Role of nasal packing in surgical outcome for chronic rhinosinusitis with polyposis. Laryngoscope.

[58] Wang J., Cai C., Wang S. (2014). Merocel versus Nasopore for nasal packing: A meta-analysis of randomized controlled trials. PLoS ONE.

[59] Fokkens W.J., Lund V.J., Hopkins C., Hellings P.W., Kern R., Reitsma S., Toppila-Salmi S., Bernal-Sprekelsen M., Mullol J. (2020). Executive summary of EPOS 2020 including integrated care pathways. Rhinology.

[60] Luong A., Ow R.A., Singh A., Weiss R.L., Han J.K., Gerencer R., Stolovitzky J.P., Stambaugh J.W., Raman A. (2018). Safety and Effectiveness of a Bioabsorbable Steroid-Releasing Implant for the Paranasal Sinus Ostia: A Randomized Clinical Trial. JAMA Otolaryngol. Head Neck Surg.

[61] US Food and Drug Administration (FDA) (2018). Premarket Approval (PMA). https://www.accessdata.fda.gov/scripts/cdrh/cfdocs/cfpma/pma.cfm.

[62] Han J.K., Kern R.C. (2019). Topical therapies for management of chronic rhinosinusitis: Steroid implants. Int. Forum Allergy Rhinol..

[63] Smith T.L., Singh A., Luong A., Ow R.A., Shotts S.D., Sautter N.B., Han J.K., Stambaugh J., Raman A. (2016). Randomized controlled trial of a bioabsorbable steroid-releasing implant in the frontal sinus opening. Laryngoscope.

[64] Forwith K.D., Han J.K., Stolovitzky J.P., Yen D.M., Chandra R.K., Karanfilov B., Matheny K.E., Stambaugh J.W., Gawlicka A.K. (2016). RESOLVE: Bioabsorbable steroid-eluting sinus implants for in-office treatment of recurrent sinonasal polyposis after sinus surgery: 6-month outcomes from a randomized, controlled, blinded study. Int. Forum Allergy Rhinol..

[65] Adriaensen G., Lim K.H., Fokkens W.J. (2017). Safety and efficacy of a bioabsorbable fluticasone propionate-eluting sinus dressing in postoperative management of endoscopic sinus surgery: A randomized clinical trial. Int. Forum Allergy Rhinol..

[66] Makadia H.K., Siegel S.J. (2011). Poly Lactic-co-Glycolic Acid (PLGA) as Biodegradable Controlled Drug Delivery Carrier. Polymers.

[67] Margolis J.R. (2009). The excel stent: A good DES, but can we really stop clopidogrel after 6 months?. JACC Cardiovasc. Interv..

[68] Kanno T., Sukegawa S., Furuki Y., Nariai Y., Sekine J. (2018). Overview of innovative advances in bioresorbable plate systems for oral and maxillofacial surgery. Jpn. Dent. Sci. Rev..

[69] Sukegawa S., Kanno T., Nagano D., Shibata A., Sukegawa-Takahashi Y., Furuki Y. (2016). The Clinical Feasibility of Newly Developed Thin Flat-Type Bioresorbable Osteosynthesis Devices for the Internal Fixation of Zygomatic Fractures: Is There a Difference in Healing Between Bioresorbable Materials and Titanium Osteosynthesis?. J. Craniofac. Surg..

[70] Haugen H.J., Lyngstadaas S.P., Rossi F., Perale G. (2019). Bone grafts: Which is the ideal biomaterial?. J. Clin. Periodontol..

[71] Pinto C.M., Asprino L., de Moraes M. (2015). Chemical and structural analyses of titanium plates retrieved from patients. Int. J. Oral Maxillofac. Surg..

[72] Acero J., Calderon J., Salmeron J.I., Verdaguer J.J., Concejo C., Somacarrera M.L. (1999). The behaviour of titanium as a biomaterial: Microscopy study of plates and surrounding tissues in facial osteosynthesis. J. Cranio-Maxillofac. Surg..

[73] Lin K.Y., Bartlett S.P., Yaremchuk M.J., Grossman R.F., Udupa J.K., Whitaker L.A. (1991). An experimental study on the effect of rigid fixation on the developing craniofacial skeleton. Plast. Reconstr. Surg..

[74] Cutright D.E., Hunsuck E.E., Beasley J.D. (1971). Fracture reduction using a biodegradable material, polylactic acid. J. Oral Surg..

[75] Park Y.-W. (2015). Bioabsorbable osteofixation for orthognathic surgery. Maxillofac. Plast. Reconstr. Surg..

[76] Suuronen R., Kallela I., Lindqvist C. (2000). Bioabsorbable plates and screws: Current state of the art in facial fracture repair. J. Craniomaxillofac. Trauma.

[77] Cural Ü., Atalay B., Yildirim M.S. (2018). Comparison of Mechanical Stabilization of the Mandibular Angulus Fracture Fixation, With Titanium Plates and Screws, Resorbable Plates and Screws, and Bone Adhesives. J. Craniofac. Surg..

[78] Sukegawa S., Kanno T., Matsumoto K., Sukegawa-Takahashi Y., Masui M., Furuki Y. (2018). Complications of a poly-L-lactic acid and polyglycolic acid osteosynthesis device for internal fixation in maxillofacial surgery. Odontology.

[79] On S.-W., Cho S.-W., Byun S.-H., Yang B.-E. (2020). Bioabsorbable Osteofixation Materials for Maxillofacial Bone Surgery: A Review on Polymers and Magnesium-Based Materials. Biomedicines.

[80] Sukegawa S., Kawai H., Nakano K., Kanno T., Takabatake K., Nagatsuka H., Furuki Y. (2019). Feasible Advantage of Bioactive/Bioresorbable Devices Made of Forged Composites of Hydroxyapatite Particles and Poly-L-lactide in Alveolar Bone Augmentation: A Preliminary Study. Int. J. Med. Sci..

[81] Schumann P., Lindhorst D., Wagner M.E.H., Schramm A., Gellrich N.C., Rücker M. (2013). Perspectives on Resorbable Osteosynthesis Materials in Craniomaxillofacial Surgery. Pathobiology.

[82] Lambotte A. (1932). L’utilisation du magnesium comme materiel perdu dans l’osteosynthèse. Bull. Mem. Soc. Nat. Chir..

[83] Leonhardt H., Franke A., McLeod N.M.H., Lauer G., Nowak A. (2017). Fixation of fractures of the condylar head of the mandible with a new magnesium-alloy biodegradable cannulated headless bone screw. Br. J. Oral Maxillofac. Surg..

[84] Shegarfi H., Reikeras O. (2009). Review article: Bone transplantation and immune response. J. Orthop. Surg..

[85] Kloss F.R., Offermanns V., Kloss-Brandstätter A. (2018). Comparison of allogeneic and autogenous bone grafts for augmentation of alveolar ridge defects-A 12-month retrospective radiographic evaluation. Clin. Oral Implant. Res..

[86] Sohn H.S., Oh J.K. (2019). Review of bone graft and bone substitutes with an emphasis on fracture surgeries. Biomater. Res..

[87] Caballé-Serrano J., Fujioka-Kobayashi M., Bosshardt D.D., Gruber R., Buser D., Miron R.J. (2016). Pre-coating deproteinized bovine bone mineral (DBBM) with bone-conditioned medium (BCM) improves osteoblast migration, adhesion, and differentiation in vitro. Clin. Oral Investig..

[88] Katz J., Mukherjee N., Cobb R.R., Bursac P., York-Ely A. (2009). Incorporation and immunogenicity of cleaned bovine bone in a sheep model. J. Biomater Appl..

[89] Cho J.S., Kim H.-S., Um S.-H., Rhee S.-H. (2013). Preparation of a novel anorganic bovine bone xenograft with enhanced bioactivity and osteoconductivity. J. Biomed. Mater. Res. Part. B Appl. Biomater..

[90] Sanz M., Dahlin C., Apatzidou D., Artzi Z., Bozic D., Calciolari E., De Bruyn H., Dommisch H., Donos N., Eickholz P. (2019). Biomaterials and regenerative technologies used in bone regeneration in the craniomaxillofacial region: Consensus report of group 2 of the 15th European Workshop on Periodontology on Bone Regeneration. J. Clin. Periodontol..

[91] Baino F., Novajra G., Vitale-Brovarone C. (2015). Bioceramics and Scaffolds: A Winning Combination for Tissue Engineering. Front. Bioeng Biotechnol..

[92] Yahav A., Kurtzman G.M., Katzap M., Dudek D., Baranes D. (2020). Bone Regeneration: Properties and Clinical Applications of Biphasic Calcium Sulfate. Dent. Clin. N. Am..

[93] Miron R.J., Zhang Q., Sculean A., Buser D., Pippenger B.E., Dard M., Shirakata Y., Chandad F., Zhang Y. (2016). Osteoinductive potential of 4 commonly employed bone grafts. Clin. Oral Investig..

[94] Donos N., Kostopoulos L., Tonetti M., Karring T., Lang N.P. (2006). The effect of enamel matrix proteins and deproteinized bovine bone mineral on heterotopic bone formation. Clin. Oral Implant. Res..

[95] Guillaume B. (2017). Filling bone defects with beta-TCP in maxillofacial surgery: A review. Morphologie.

[96] Friedmann A., Dehnhardt J., Kleber B.M., Bernimoulin J.P. (2008). Cytobiocompatibility of collagen and ePTFE membranes on osteoblast-like cells in vitro. J. Biomed. Mater. Res. A.

[97] Rodella L.F., Favero G., Labanca M. (2011). Biomaterials in maxillofacial surgery: Membranes and grafts. Int J. Biomed. Sci.

[98] Urban I.A., Monje A. (2019). Guided Bone Regeneration in Alveolar Bone Reconstruction. Oral Maxillofac. Surg. Clin. N. Am..

[99] Jiménez Garcia J., Berghezan S., Caramês J.M.M., Dard M.M., Marques D.N.S. (2017). Effect of cross-linked vs non-cross-linked collagen membranes on bone: A systematic review. J. Periodontal. Res..

[100] Sbricoli L., Guazzo R., Annunziata M., Gobbato L., Bressan E., Nastri L. (2020). Selection of Collagen Membranes for Bone Regeneration: A Literature Review. Materials.

[101] Martin-Thomé H., Bourdin D., Strube N., Saffarzadeh A., Morlock J.F., Campard G., Evanno C., Hoornaert A., Layrolle P. (2018). Clinical Safety of a New Synthetic Resorbable Dental Membrane: A Case Series Study. J. Oral Implant..

[102] Souza F.G.R., Santos I.C., Bergmann A., Thuler L.C.S., Freitas A.S., Freitas E.Q., Dias F.L. (2020). Quality of life after total laryngectomy: Impact of different vocal rehabilitation methods in a middle income country. Health Qual. Life Outcomes.

[103] Bertl K., Zatorska B., Leonhard M., Matejka M., Schneider-Stickler B. (2012). Anaerobic and microaerophilic pathogens in the biofilm formation on voice prostheses: A pilot study. Laryngoscope.

[104] Bucki R., Niemirowicz-Laskowska K., Deptuła P., Wilczewska A.Z., Misiak P., Durnaś B., Fiedoruk K., Piktel E., Mystkowska J., Janmey P.A. (2019). Susceptibility of microbial cells to the modified PIP(2)-binding sequence of gelsolin anchored on the surface of magnetic nanoparticles. J. Nanobiotechnol..

[105] Durnaś B., Wnorowska U., Pogoda K., Deptuła P., Wątek M., Piktel E., Głuszek S., Gu X., Savage P.B., Niemirowicz K. (2016). Candidacidal Activity of Selected Ceragenins and Human Cathelicidin LL-37 in Experimental Settings Mimicking Infection Sites. PLoS ONE.

[106] Durnaś B., Piktel E., Wątek M., Wollny T., Góźdź S., Smok-Kalwat J., Niemirowicz K., Savage P.B., Bucki R. (2017). Anaerobic bacteria growth in the presence of cathelicidin LL-37 and selected ceragenins delivered as magnetic nanoparticles cargo. BMC Microbiol..

[107] Niemirowicz K., Durnaś B., Tokajuk G., Piktel E., Michalak G., Gu X., Kułakowska A., Savage P.B., Bucki R. (2017). Formulation and candidacidal activity of magnetic nanoparticles coated with cathelicidin LL-37 and ceragenin CSA-13. Sci. Rep..

[108] Niemirowicz-Laskowska K., Mystkowska J., Łysik D., Chmielewska S., Tokajuk G., Misztalewska-Turkowicz I., Wilczewska A.Z., Bucki R. (2020). Antimicrobial and Physicochemical Properties of Artificial Saliva Formulations Supplemented with Core-Shell Magnetic Nanoparticles. Int. J. Mol. Sci..

[109] Vallieres C., Hook A.L., He Y., Crucitti V.C., Figueredo G., Davies C.R., Burroughs L., Winkler D.A., Wildman R.D., Irvine D.J. (2020). Discovery of (meth)acrylate polymers that resist colonization by fungi associated with pathogenesis and biodeterioration. Sci. Adv..

[110] Niermeyer W.L., Rodman C., Li M.M., Chiang T. (2020). Tissue engineering applications in otolaryngology-The state of translation. Laryngoscope Investig. Otolaryngol..

[111] Cao Y., Vacanti J.P., Paige K.T., Upton J., Vacanti C.A. (1997). Transplantation of Chondrocytes Utilizing a Polymer-Cell Construct to Produce Tissue-Engineered Cartilage in the Shape of a Human Ear. Plast. Reconstr. Surg..

[112] Fulco I., Miot S., Haug M.D., Barbero A., Wixmerten A., Feliciano S., Wolf F., Jundt G., Marsano A., Farhadi J. (2014). Engineered autologous cartilage tissue for nasal reconstruction after tumour resection: An observational first-in-human trial. Lancet.

[113] Hoshi K., Fujihara Y., Saijo H., Kurabayashi K., Suenaga H., Asawa Y., Nishizawa S., Kanazawa S., Uto S., Inaki R. (2017). Three-dimensional changes of noses after transplantation of implant-type tissue-engineered cartilage for secondary correction of cleft lip–nose patients. Regen. Ther..

[114] Zhou G., Jiang H., Yin Z., Liu Y., Zhang Q., Zhang C., Pan B., Zhou J., Zhou X., Sun H. (2018). In Vitro Regeneration of Patient-specific Ear-shaped Cartilage and Its First Clinical Application for Auricular Reconstruction. EBioMedicine.

[115] Nimeskern L., Martínez Ávila H., Sundberg J., Gatenholm P., Müller R., Stok K.S. (2013). Mechanical evaluation of bacterial nanocellulose as an implant material for ear cartilage replacement. J. Mech. Behav. Biomed. Mater..

[116] Lee M.C., Seonwoo H., Garg P., Jang K.J., Pandey S., Park S.B., Kim H.B., Lim J., Choung Y.H., Chung J.H. (2018). Chitosan/PEI patch releasing EGF and the EGFR gene for the regeneration of the tympanic membrane after perforation. Biomater. Sci..

[117] Mellott A.J., Shinogle H.E., Nelson-Brantley J.G., Detamore M.S., Staecker H. (2017). Exploiting decellularized cochleae as scaffolds for inner ear tissue engineering. Stem Cell Res. Ther..

[118] Maughan E.F., Butler C.R., Crowley C., Teoh G.Z., den Hondt M., Hamilton N.J., Hynds R.E., Lange P., Ansari T., Urbani L. (2017). A comparison of tracheal scaffold strategies for pediatric transplantation in a rabbit model. Laryngoscope.

[119] Zhao L., Sundaram S., Le A.V., Huang A.H., Zhang J., Hatachi G., Beloiartsev A., Caty M.G., Yi T., Leiby K. (2016). Engineered Tissue-Stent Biocomposites as Tracheal Replacements. Tissue Eng. Part A.

[120] Dharmadhikari S., Liu L., Shontz K., Wiet M., White A., Goins A., Akula H., Johnson J., Reynolds S.D., Breuer C.K. (2020). Deconstructing tissue engineered trachea: Assessing the role of synthetic scaffolds, segmental replacement and cell seeding on graft performance. Acta Biomate.r.

[121] Best C.A., Pepper V.K., Ohst D., Bodnyk K., Heuer E., Onwuka E.A., King N., Strouse R., Grischkan J., Breuer C.K. (2018). Designing a tissue-engineered tracheal scaffold for preclinical evaluation. Int. J. Pediatr. Otorhinolaryngol..

[122] Brookes S., Voytik-Harbin S., Zhang H., Zhang L., Halum S. (2019). Motor endplate-expressing cartilage-muscle implants for reconstruction of a denervated hemilarynx. Laryngoscope.

[123] Herrmann P., Ansari T., Southgate A., Varanou Jenkins A., Partington L., Carvalho C., Janes S., Lowdell M., Sibbons P.D., Birchall M.A. (2019). In vivo implantation of a tissue engineered stem cell seeded hemi-laryngeal replacement maintains airway, phonation, and swallowing in pigs. J. Tissue Eng. Regen. Med..

[124] Olsen L.B., Larsen S., Wanscher J.H., Faber C.E., Jeppesen J. (2018). Postoperative infections following cochlear implant surgery. Acta Otolaryngol..

[125] Mikulskis P., Hook A., Dundas A.A., Irvine D., Sanni O., Anderson D., Langer R., Alexander M.R., Williams P., Winkler D.A. (2018). Prediction of Broad-Spectrum Pathogen Attachment to Coating Materials for Biomedical Devices. ACS Appl. Mater. Interfaces.

[126] Wei B.P., Shepherd R.K., Robins-Browne R.M., Clark G.M., O’Leary S.J. (2010). Pneumococcal meningitis post-cochlear implantation: Potential routes of infection and pathophysiology. Otolaryngol. Head Neck Surg..

[127] Kirchhoff L., Arweiler-Harbeck D., Arnolds J., Hussain T., Hansen S., Bertram R., Buer J., Lang S., Steinmann J., Höing B. (2020). Imaging studies of bacterial biofilms on cochlear implants-Bioactive glass (BAG) inhibits mature biofilm. PLoS ONE.

[128] Vargas-Blanco D., Lynn A., Rosch J., Noreldin R., Salerni A., Lambert C., Rao R.P. (2017). A pre-therapeutic coating for medical devices that prevents the attachment of Candida albicans. Ann. Clin. Microbiol. Antimicrob..

[129] Kao W.K., Gagnon P.M., Vogel J.P., Chole R.A. (2017). Surface charge modification decreases *Pseudomonas aeruginosa* adherence in vitro and bacterial persistence in an in vivo implant model. Laryngoscope.

[130] Chen R., Willcox M.D., Ho K.K., Smyth D., Kumar N. (2016). Antimicrobial peptide melimine coating for titanium and its in vivo antibacterial activity in rodent subcutaneous infection models. Biomaterials.

[131] Höing B., Kirchhoff L., Arnolds J., Hussain T., Buer J., Lang S., Arweiler-Harbeck D., Steinmann J. (2018). Bioactive Glass Granules Inhibit Mature Bacterial Biofilms on the Surfaces of Cochlear Implants. Otol. Neurotol..

[132] Parent M., Magnaudeix A., Delebassée S., Sarre E., Champion E., Viana Trecant M., Damia C. (2016). Hydroxyapatite microporous bioceramics as vancomycin reservoir: Antibacterial efficiency and biocompatibility investigation. J. Biomater. Appl..

[133] Lim D.J., Skinner D., Mclemore J., Rivers N., Elder J.B., Allen M., Koch C., West J., Zhang S., Thompson H.M. (2020). In-vitro evaluation of a ciprofloxacin and azithromycin sinus stent for *Pseudomonas aeruginosa* biofilms. Int Forum Allergy Rhinol..

[134] Şevik Eliçora S., Erdem D., Dinç A.E., Altunordu Kalaycı Ö., Hazer B., Yurdakan G., Külah C. (2017). Effects of polymer-based, silver nanoparticle-coated silicone splints on the nasal mucosa of rats. Eur. Arch. Otorhinolaryngol..

[135] Danti S., Azimi B., Candito M., Fusco A., Sorayani Bafqi M.S., Ricci C., Milazzo M., Cristallini C., Latifi M., Donnarumma G. (2020). Lithium niobate nanoparticles as biofunctional interface material for inner ear devices. Biointerphases.

[136] Edmondson S., Osborne V.L., Huck W.T.S. (2004). Polymer brushes via surface-initiated polymerizations. Chem. Soc. Rev..

[137] Kabirian F., Ditkowski B., Zamanian A., Hoylaerts M.F., Mozafari M., Heying R. (2019). Controlled NO-Release from 3D-Printed Small-Diameter Vascular Grafts Prevents Platelet Activation and Bacterial Infectivity. ACS Biomater. Sci. Eng..

[138] Gao Q., Yu M., Su Y., Xie M., Zhao X., Li P., Ma P.X. (2017). Rationally designed dual functional block copolymers for bottlebrush-like coatings: In vitro and in vivo antimicrobial, antibiofilm, and antifouling properties. Acta Biomater..

[139] Cheng Q., Asha A.B., Liu Y., Peng Y.-Y., Diaz-Dussan D., Shi Z., Cui Z., Narain R. (2021). Antifouling and Antibacterial Polymer-Coated Surfaces Based on the Combined Effect of Zwitterions and the Natural Borneol. ACS Appl. Mater. Interfaces.

[140] Kurowska M., Eickenscheidt A., Guevara-Solarte D.-L., Widyaya V.T., Marx F., Al-Ahmad A., Lienkamp K. (2017). A Simultaneously Antimicrobial, Protein-Repellent, and Cell-Compatible Polyzwitterion Network. Biomacromolecules.

[141] Qiu H., Si Z., Luo Y., Feng P., Wu X., Hou W., Zhu Y., Chan-Park M.B., Xu L., Huang D. (2020). The Mechanisms and the Applications of Antibacterial Polymers in Surface Modification on Medical Devices. Front. Bioeng. Biotechnol..

[142] Wong S.Y., Han L., Timachova K., Veselinovic J., Hyder M.N., Ortiz C., Klibanov A.M., Hammond P.T. (2012). Drastically Lowered Protein Adsorption on Microbicidal Hydrophobic/Hydrophilic Polyelectrolyte Multilayers. Biomacromolecules.

[143] Meng S., Liu Z., Shen L., Guo Z., Chou L.L., Zhong W., Du Q., Ge J. (2009). The effect of a layer-by-layer chitosan–heparin coating on the endothelialization and coagulation properties of a coronary stent system. Biomaterials.

[144] Li D., Dai F., Li H., Wang C., Shi X., Cheng Y., Deng H. (2021). Chitosan and collagen layer-by-layer assembly modified oriented nanofibers and their biological properties. Carbohydr. Polym..

[145] Vaterrodt A., Thallinger B., Daumann K., Koch D., Guebitz G.M., Ulbricht M. (2016). Antifouling and Antibacterial Multifunctional Polyzwitterion/Enzyme Coating on Silicone Catheter Material Prepared by Electrostatic Layer-by-Layer Assembly. Langmuir.

[146] Ghamrawi S., Bouchara J.-P., Tarasyuk O., Rogalsky S., Lyoshina L., Bulko O., Bardeau J.-F. (2017). Promising silicones modified with cationic biocides for the development of antimicrobial medical devices. Mater. Sci. Eng. C.

[147] Dirain C.O., Silva R.C., Antonelli P.J. (2016). Prevention of biofilm formation by polyquaternary polymer. Int. J. Pediatric Otorhinolaryngol..

[148] Rossi S., Azghani A.O., Omri A. (2004). Antimicrobial efficacy of a new antibiotic-loaded poly(hydroxybutyric-co-hydroxyvaleric acid) controlled release system. J. Antimicrob. Chemother..

[149] Pritchard E.M., Valentin T., Panilaitis B., Omenetto F., Kaplan D.L. (2013). Antibiotic-Releasing Silk Biomaterials for Infection Prevention and Treatment. Adv. Funct. Mater..

[150] Marsili L., Dal Bo M., Berti F., Toffoli G. (2021). Chitosan-Based Biocompatible Copolymers for Thermoresponsive Drug Delivery Systems: On the Development of a Standardization System. Pharmaceutics.

[151] Divya K.P., Miroshnikov M., Dutta D., Vemula P.K., Ajayan P.M., John G. (2016). In Situ Synthesis of Metal Nanoparticle Embedded Hybrid Soft Nanomaterials. Acc. Chem. Res..

[152] Sharma V.K., Yngard R.A., Lin Y. (2009). Silver nanoparticles: Green synthesis and their antimicrobial activities. Adv. Colloid Interface Sci..

[153] Ma L., Li K., Xia J., Chen C., Liu Y., Lang S., Yu L., Liu G. (2022). Commercial soft contact lenses engineered with zwitterionic silver nanoparticles for effectively treating microbial keratitis. J. Colloid Interface Sci..

[154] Ye Z., Sang T., Li K., Fischer N.G., Mutreja I., Echeverría C., Kumar D., Tang Z., Aparicio C. (2022). Hybrid nanocoatings of self-assembled organic-inorganic amphiphiles for prevention of implant infections. Acta Biomater..

[155] Duda F., Bradel S., Bleich A., Abendroth P., Heemeier T., Ehlert N., Behrens P., Esser K.H., Lenarz T., Brandes G. (2015). Biocompatibility of silver containing silica films on Bioverit® II middle ear prostheses in rabbits. J. Biomater. Appl..

[156] Jang C.H., Cho Y.B., Jang Y.S., Kim M.S., Kim G.H. (2015). Antibacterial effect of electrospun polycaprolactone/polyethylene oxide/vancomycin nanofiber mat for prevention of periprosthetic infection and biofilm formation. Int. J. Pediatr. Otorhinolaryngol..

[157] Ziabka M., Dziadek M., Krolicka A. (2019). Biological and Physicochemical Assessment of Middle Ear Prosthesis. Polymers.

[158] Ziabka M., Dziadek M., Menaszek E., Banasiuk R., Krolicka A. (2017). Middle Ear Prosthesis with Bactericidal Efficacy-In Vitro Investigation. Molecules.

[159] Barros J., Grenho L., Fernandes M.H., Manuel C.M., Melo L.F., Nunes O.C., Monteiro F.J., Ferraz M.P. (2015). Anti-sessile bacterial and cytocompatibility properties of CHX-loaded nanohydroxyapatite. Colloids Surf. B Biointerfaces.

[160] Weng W., Li X., Nie W., Liu H., Liu S., Huang J., Zhou Q., He J., Su J., Dong Z. (2020). One-Step Preparation of an AgNP-nHA@RGO Three-Dimensional Porous Scaffold and Its Application in Infected Bone Defect Treatment. Int. J. Nanomed..

[161] Wang Q., Tang Y., Ke Q., Yin W., Zhang C., Guo Y., Guan J. (2020). Magnetic lanthanum-doped hydroxyapatite/chitosan scaffolds with endogenous stem cell-recruiting and immunomodulatory properties for bone regeneration. J. Mater. Chem. B.

[162] Wang J., Zhou H., Guo G., Tan J., Wang Q., Tang J., Liu W., Shen H., Li J., Zhang X. (2017). Enhanced Anti-Infective Efficacy of ZnO Nanoreservoirs through a Combination of Intrinsic Anti-Biofilm Activity and Reinforced Innate Defense. ACS Appl. Mater. Interfaces.

[163] Chen B., You Y., Ma A., Song Y., Jiao J., Song L., Shi E., Zhong X., Li Y., Li C. (2020). Zn-Incorporated TiO. Int. J. Nanomed..

